# Predictiveness of Disease Risk in a Global Outreach Tourist Setting in Thailand Using Meteorological Data and Vector-Borne Disease Incidences

**DOI:** 10.3390/ijerph111010694

**Published:** 2014-10-16

**Authors:** Suwannapa Ninphanomchai, Chitti Chansang, Yien Ling Hii, Joacim Rocklöv, Pattamaporn Kittayapong

**Affiliations:** 1 Center of Excellence for Vectors and Vector-Borne Diseases, Faculty of Science, Mahidol University at Salaya, Nakhon Pathom 73170, Thailand; E-Mail: ninphanomchai@gmail.com; 2 Department of Medical Sciences; Ministry of Public Health, Nonthaburi 11000, Thailand; E-Mail: chittichan@hotmail.com; 3 Umeå Centre for Global Health Research, Department of Public Health and Clinical Medicine, Epidemiology and Global Health, Umeå University, Umeå 90187, Sweden; E-Mails: yienling.hii@epiph.umu.se (Y.L.H.); joacim.rocklov@epiph.umu.se (J.R.)

**Keywords:** climatic factors, dengue, malaria, Thailand, vector-borne diseases

## Abstract

Dengue and malaria are vector-borne diseases and major public health problems worldwide. Changes in climatic factors influence incidences of these diseases. The objective of this study was to investigate the relationship between vector-borne disease incidences and meteorological data, and hence to predict disease risk in a global outreach tourist setting. The retrospective data of dengue and malaria incidences together with local meteorological factors (temperature, rainfall, humidity) registered from 2001 to 2011 on Koh Chang, Thailand were used in this study. Seasonal distribution of disease incidences and its correlation with local climatic factors were analyzed. Seasonal patterns in disease transmission differed between dengue and malaria. Monthly meteorological data and reported disease incidences showed good predictive ability of disease transmission patterns. These findings provide a rational basis for identifying the predictive ability of local meteorological factors on disease incidence that may be useful for the implementation of disease prevention and vector control programs on the tourism island, where climatic factors fluctuate.

## 1. Introduction

Dengue and malaria are vector-borne diseases that pose a major public health problem in many tropical countries. Both diseases cause infections in more than 100 million people each year in over 100 countries [1,2]. Environmental, socio-economic, and climatic factors (temperature, humidity and rainfall) affect disease infection and influence transmission patterns of these diseases [3,4,5]. In Thailand, dengue and malaria incidences were estimated to be 124.60 and 25.20 per 100,000 population, respectively, in 2012 [6]. In addition, from January–August 2013, about 99,452 cases with 94 deaths and 9,419 cases with six deaths were reported for dengue and malaria, respectively (morbidity rate = 154.75 for dengue and 14.66/100,000 population for malaria) [7]. According to the Provincial Public Health Office of Trat Province, dengue and malaria were the most common vector-borne diseases reported on Koh Chang, where the morbidity rate of malaria and dengue were 105.00 and 68.63 per 100,000 populations respectively (Trat Province had the 4th highest incidence rate in Thailand). Thereby, the predictiveness of dengue and malaria in this tourist setting area needs to be investigated.

Dengue and malaria are well-known as climate-sensitive diseases for many reasons. In many tropical regions, temperature and rainfall levels contribute to the presence of active adult vectors throughout the year, which enables a continuous transmission cycle and makes the diseases endemic [8]. A rise in temperature accelerates the metabolic rate of a vector, increases biting rate, enhances more frequent blood feeding rates, and also favors egg production and increases in population size [9]. Moreover, high relative humidity is beneficial for most metabolic processes in vectors. A combination of high temperature and high humidity will prolong the survival of many arthropod vectors. But in low humidity conditions, sometimes blood feeding rates could increase, as vectors attempt to compensate for the high level of water loss [9]. Rainfall correlates positively to the presence of breeding sites, but heavy or prolonged rain may disrupt the breeding sites by washing away the immature stages or killing them directly [9].

For dengue, its transmission is a seasonal pattern that punctuates every few years. Changes in temperature and precipitation have well-known roles in changing incidence levels of dengue via altering its transmission cycle [10]. The association between climate and dengue virus largely depends on local climate and, thus, the patterns vary among geographical areas [9,10,11]. However, impact of local climate on vector-borne disease incidence is less understood, with only limited scientific evidence reported and no consensus on the main drivers.

Dengue and malaria are the most important vector-borne diseases on Koh Chang. No previous study had elaborated the association and predictiveness of meteorological patterns on vector-borne diseases in this tourism setting, where heavy rainfall occurs year round. As both dengue and malaria are climatically sensitive and their spread is influenced or favored by temporal effects, investigation of the relationship between these diseases and local meteorological factors can be challenging. Koh Chang is a popular tourist destination for both Thai and foreign visitors, and the number of tourists coming to this island increase each year; so the transmission that occurs on the island may not only create public health problems on the local scale but also nationally and internationally. Therefore, the predictive ability of local meteorological factors on vector-borne disease incidence (dengue and malaria) on Koh Chang (which serves as an example of a global outreach tourist destination) remains in question and needs to be examined.

In this study, we aim to explore the impact of local meteorological factors on the temporal distribution of dengue and malaria on Koh Chang. Local meteorological data (temperature, rainfall, humidity) coupled with epidemiological data (dengue and malaria incidences) registered from 2001 to 2011 on Koh Chang were analyzed at different time scales to explore their relationship.

**Table 1:** Climate conditions classified by month and season on Koh Chang, 2001–2011.

Month	Temperature (°C)	Rainy (Days)	Rainfall (mm)	Relative Humidity (%)
Jan	26.7	4.3	34.1	71.0
Feb	27.2	17.4	102.0	76.8
Mar	27.9	14.5	162.2	78.6
Apr	28.5	17.5	211.5	80.2
May	28.3	23.1	489.1	82.9
Jun	27.6	25.5	692.9	85.3
Jul	27.1	27.0	1057.2	86.3
Aug	27.1	27.1	879.1	86.5
Sep	27.1	24.4	835.4	86.3
Oct	27.3	19.9	403.6	83.5
Nov	27.7	7.9	53.7	73.4
Dec	27.1	3.1	31.4	68.4
Total	27.5 ± 0.7	16.8 ± 9.3	412.7 ± 425.7	79.9 ± 6.5

Note: ^a^ Data are monthly cumulative.

## 2. Methods

### 2.1. Study Area

This study was conducted on Koh Chang, a district of Trat Province in the eastern part of Thailand, located 330 kilometers from Bangkok. The name “Koh Chang” means Elephant Island and was adopted because the shape of the island looks like an elephant head. It is the largest island in the Gulf of Thailand and a popular tourism destination for both local and international tourists. The island is located between latitude 11°33′N–12°15′N and longitude 102°15′–102°55′ E. The climate is influenced by the South Western Monsoon season, with heavy rainfall occurring especially between May and October. Average rainfall is 408.54 mm (2001–2011) with an average temperature of 27.47 °C and a relative humidity of 79.91% (Table 1). Koh Chang District occupies an area of 154.8 km^2^ with a plain elevation more than 10–70 meters above sea level. From 2003 to 2011, the registered population of Koh Chang District was between 4970 and 7671, with a total of 2,177 to 4,642 households [12]. Population density on Koh Chang was estimated to be from 1:32.1 to 1:49.5 people per km^2^ [13], and more than half of the inhabitants are concentrated in the western part of the island. Administratively, Koh Chang is divided into two sub-districts; (1) Koh Chang Subdistrict, including Klong Non Sri, Dan Mai, Klong Son, and Klong Prao villages; and (2) Koh Chang Tai Subdistrict, including Bang Bao, Saluk Phet, Jek Bae, Saluk Khok, and Saluk Phet Nheu villages.

### 2.2. Epidemiological Data

Daily reported cases of dengue and malaria from 2001–2011 were used in this study. Clinically suspected cases of dengue and confirmed malaria reported cases by using blood smear test were investigated. The reported cases of malaria in 2005 were missing and excluded from this study. The data was collected by the Center for Disease Control, the Provincial Public Health Office of Trat Province. The data were obtained from the R506 report, which uses a code number such as 26—Dengue Hemorrhagic fever (DHF), 27—Dengue Shock Syndrome (DSS), 66—Dengue Fever (DF), and 30—Malaria. The form 506 provided data for each patient’s age, gender, address, occupation, race, date of admission, and date of recovery. Each daily reported case was then compiled into a monthly report.

### 2.3. Meteorological Data

Meteorological data registered daily from the period of 2001–2011, including rainfall (mm), temperature (°C) and relative humidity (%), were used in this study. The measurement of relative humidity was partly influenced by air temperature and water or rain. We removed these influences through regression process and used the residuals (res_hum), which were not explained by temperature and rainfall, as a variable to replace relative humidity in the model development.

There was one rain station situated on Koh Chang but no regular data was registered and there was missing information, so our main resource of climatic data was recorded by Trat Meteorological Station (Station code 501201), provided by the Thai Meteorological Department (TMD), Ministry of Information and Communication Technology, Thailand. This weather station is the nearest weather station providing observations that are representative of the local climate around Koh Chang. It is situated in Khlong Yai District, located in the mainland at 11°46'49.2"N and 102°52'41.1"E, about 15 km from Koh Chang.

### 2.4. Statistical Analysis

We developed Poisson regression models to analyze the relationship between meteorological predictors (independent variables) and malaria/dengue cases (dependent variables) over the past decade. Quasi-Poisson was chosen to allow over-dispersion of count data. The model development process was comprised of data examination, model identification, estimation, selection, and validation. We modeled the data based on statistical analysis and prior information on general knowledge of the vector’s life cycle and disease transmission.

We examined the time series distribution patterns of each variable based on graphical presentations, including epidemiologic curves, histograms, and scatter plots. Statistical functions such as Autocorrelation (ACF) and partial autocorrelation functions (PACF) were used to study the relationship between past and current cases. The PACF of the diseases suggested that malaria and dengue cases reported in the current month could be influenced by the cases reported in the previous one and two months, respectively. We included cases in the past months as independent variables to account for all the effects not explained by the meteorological variables. It has been documented that the influence of climate on vector-borne diseases (as is translated through its impacts on the life cycles of vectors) is responsible for disease transmission. Cross correlation analysis showed that there was a time lag between exposure to favorable meteorological conditions and responses or occurrence and the reporting of disease cases. To account for this effect, we created up to seven lag terms at an interval of one month for each independent variable. We further applied cubic spline smoothing on each independent variable to permit analysis of a non-linear relationship between exposure and the responses. Also, mid-year population was included to account for changes of cases influenced by the size of population in the study area.

For each of the two diseases, we developed multiple models using a general additive model (GAM) in R statistics and computing software. These models comprised of meteorological factors with different combination of lag terms ranging from 0 to 7. Selection criteria of an optimal model for each disease was based on the lowest generalized cross validation scores (GCV), highest coefficient of determinant (R^2^) or a number that indicates how well a model fits the dataset, and lowest standardized root mean square errors (SRMSE) or errors between fitted and reported cases. We developed a series of models ranging from bivariate and multivariate, using a different combination of meteorological variables with lag terms ranging from single to combined number of terms. We then simulated each of these models with and without autoregressive terms to compare the results and percent of deviance explained by each model. Each regression model began with full model and lag terms that were not statistically significant based on 95% confidence levels (*i.e.*, not related to the disease distribution according to the statistical analysis) were removed one at a time until the model reached optimal GCV scores, R^2^ and SRMSE. Finally, we select the optimal models based on simplicity of the model and fulfillment of selection criteria, and good fit of the model on dataset. To determine the fitness of the model or the sufficiency of each model to interpret the dataset, we validated each model through residual diagnoses, which included histogram, PACF, residual plot, and a time series plot of fitted against reported cases to analyze the normality, linearity, and independence of the fitted data. Statistical analyses were performed using STATA 12 (StataCorp, Texas, LP, USA) and R 3.0.1 (R Project for Statistical Computing).

The optimal models are presented as follows:

1. Dengue Model

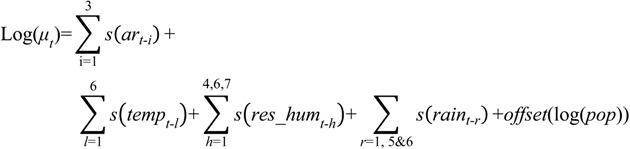
(1)
where *log(μ_t_)* represents the average predicted number of cases; s depicts cubic spline smoothing with 3 degrees of freedom; and *i*, *l*, *h*, *r*, are the lag terms in month(s) corresponding to each respective variable. Note that *h* = 5 or lag term 5 of *res_hum* was removed; *ar* denotes auto-regression terms of past cases; *temp* stands for mean temperature; *res_hum* represents the adjusted relative humidity; *rain* equates rainfall; and *pop* depicts mid-year population.

2. Malaria model

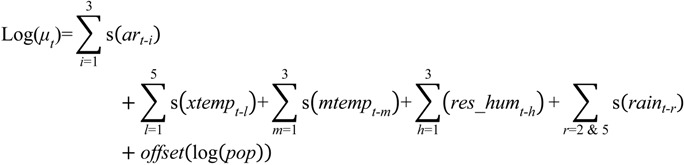
(2)
where *log (μ_t_)* represents the average predicted number of cases; s depicts cubic spline smoothing with 3 degrees of freedom; *i*, *l*, *m*, *h*, *r* are the lag terms in month(s) for each respective variable; *ar* denotes auto-regression terms of past cases; *xtemp* stands for maximum temperature; *mtemp* is the minimum temperature; *res_hum* represents the adjusted relative humidity; *rain* equates rainfall; and *pop* depicts mid-year population.

## 3. Results

### 3.1. Dengue

#### 3.1.1. Seasonal Variation and Dengue Incidence

**Figure 1:**
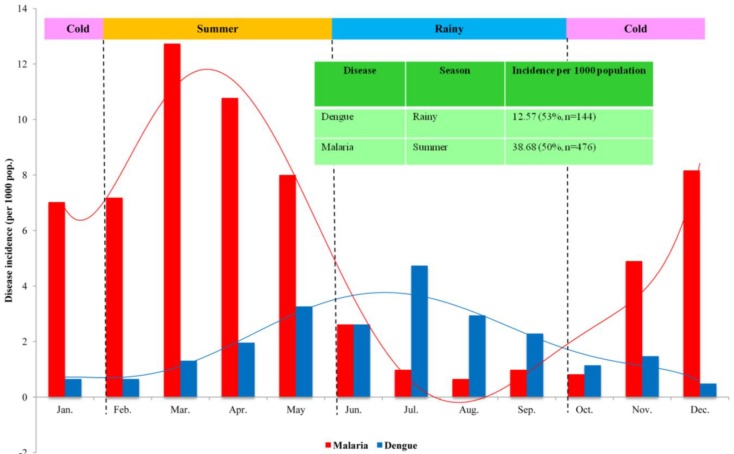
Number of dengue and malaria cases reported on monthly basis on Koh Chang during the years 2001–2011.

From a total of 144 dengue cases reported from 2001 to 2011 on Koh Chang, dengue was highly present in the rainy season (June-September) (53.47%, n = 77), followed by summer (February–May) (30.56%, n = 44), and the cold season (October-January) (15.97%, n = 23). Figure 1 illustrates monthly dengue incidence over the 11 years of study. Dengue incidence ranged from 0.49 to 4.73 per 1000 population (1.96 ± 1.27) over the 11 years of study. It was highest in the rainy season then decreasing during the cold season but starting to increase in the summer season until reaching its highest peak in the rainy season then the cycle continued all year round. July was the month with the highest incidence (4.73 per 1000 pop.) followed by May (3.27 per 1000 pop.) and August (2.94 per 1000 pop.). The lowest incidence was in December (0.49 per 1000 pop.). During the cold season, dengue incidence started to decrease; then it increased during the summer, reaching its highest peak during the rainy season. Dengue tended to occur in the period with the highest rainfall (rainy season: June–September); its distribution might follow the rainfall trend. Thereby, changes in rainfall patterns or the amount of rainfall might affect dengue transmission.

#### 3.1.2 Correlation between Climatic Factors and Dengue Incidence

Pearson correlation tests were used to investigate the correlation between climatic factors (temperature, rainfall, humidity) and disease incidence. Minimum temperature, mean temperature, rainfall, and relative humidity were positively correlated with dengue incidence. Maximum temperature, was usually positively correlated with dengue incidence, except at t-0 and t-2. The highest correlation between dengue incidence and maximum temperature, minimum temperature, mean temperature, rainfall, and relative humidity was 0.155; 0.194; 0.185; 0.096; and 0.207 at t-10, t-2; t-10; t-0, and t-5 respectively. All were statistically significant at a 0.01 level (*p* < 0.01).

#### 3.1.3. Empirical Model of Climatic Factors and Dengue Incidence

**Figure 2:**
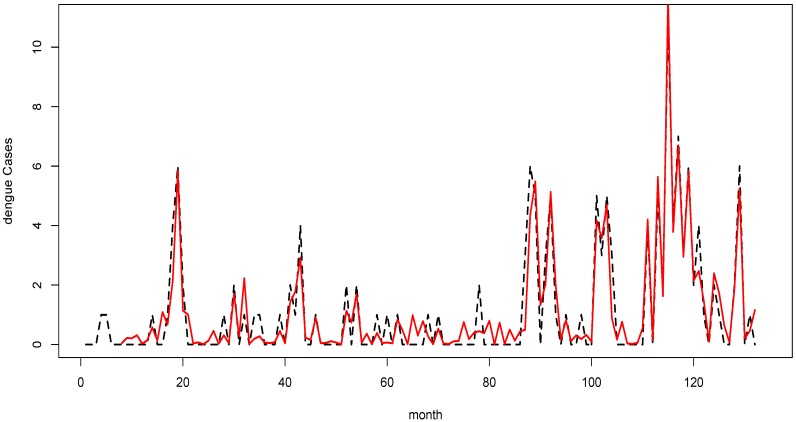
Reported dengue cases (black dotted line) and fitted cases (red line) generated by model for the years 2001–2011.

The results based on model (1) suggested that adjusted relative humidity preceded dengue incidence by up to seven months, while mean temperature and rainfall led dengue incidence by up to six months. Overall, the model explained about 80% of the distribution of dengue incidence. While, the model explained about 50% of the disease distribution without the influence of past cases. Residual diagnoses suggested that the model was sufficient for interpretation. Residual plots suggested data were random, linear, and almost normal. PACF showed all spikes were contained within the upper and lower bounds of confidence intervals. The time series plot of fitted cases against reported dengue cases further suggested a fit with the model (Figure 2).

**Figure 3:**
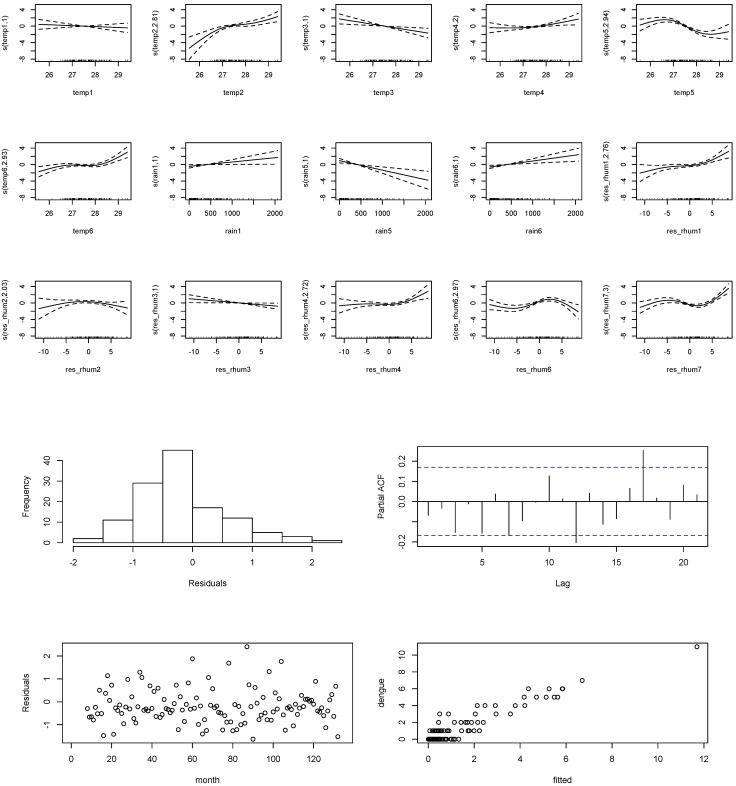
Relative risk of dengue cases as functions of respective climate variables at various lag times.

As shown in Figure 3, mean temperature preceded risk of dengue by two to six months. Each unit increase in mean temperature above 27 °C increased the relative risk of dengue almost linearly at lag of two and four months; while temperatures above 28 °C posed a similar threat at a longer lag of 6 months. Conversely, mean temperature showed an inverse positive relationship with dengue at a lag of three and five months. Also, the results showed a positive and linear increase in the relative risk of dengue at a lag of one and six months after each unit increase in rainfall. Low rainfall from 0 mm posed a high risk of dengue at the lag of five months, with reduced risk corresponding to each unit surge in rainfall up to 500 mm. The adjusted relative humidity or properties in relative humidity not explained by temperature and rainfall posed a threat to the increase in relative risk of dengue at a lag months one to seven, with highest risk occurring at a lag of one, four and seven months.

### 3.2. Malaria

#### 3.2.1. Seasonal Variation and Malaria Incidence

From a total of 476 cases reported from 2001 to 2011 on Koh Chang, malaria was highly present in the summer (March–May) (61.55%, n = 293), followed by the cold season (October–February) (29.83%, n = 142), and dropping during the rainy season (June–September) (8.61%, n = 41). Figure 1 illustrates monthly malaria incidence over the 11 years of study. It was highest in the summer season then decreasing during the rainy season but starting to increase in the cold season until reaching its highest in the summer season then the cycle continued all year round. Malaria incidence ranged from 0.65 to 12.73 per 1000 population (pop.) (5.40 ± 4.19) over the 11 years of study. March was the month with the highest incidence (12.73 per 1000 pop.) followed by April (10.76 per 1000 pop.) and May (8.0 per 1000 pop.). The lowest incidence of malaria was in August (0.65 per 1000 pop.). During the cold season, malaria incidence started to increase, reaching its highest peak during summer, and was followed by a decrease during the rainy season. Malaria seemed to be reported in the period with less rainfall (summer: February–May). Therefore, malaria distribution might conversely follow the rainfall trend. Change in rainfall pattern or amount of rainfall might conversely affect malaria transmission.

#### 3.2.2. Correlation between Meteorological Factors and Malaria Incidence

Rainfall and average humidity were negatively correlated with malaria incidence. Maximum temperature was positively correlated with malaria incidence, except at t-10. Minimum temperature was mostly negatively correlated with malaria incidence, except at t-0 and t-1. Mean temperature was mostly positively correlated with malaria incidence, except at t-6 to t-10. The highest correlation between malaria incidence and maximum temperature, minimum temperature, mean temperature, rainfall, and relative humidity was 0.150; −0.233; 0.190; −0.183; and −0.190 at t-1; t-10; t-1; t-9, and t-10 respectively. All were statistically significant at a 0.01 level (*p* < 0.01).

#### 3.2.3. Empirical Model of Meteorological Factors and Malaria Incidence

**Figure 4:**
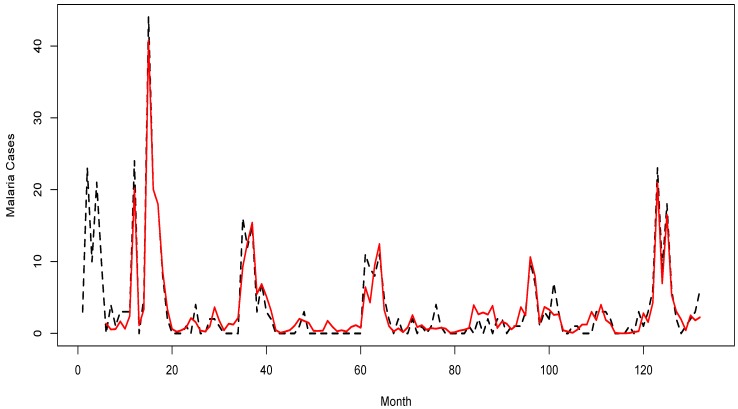
Reported malaria cases (black dotted line) and fitted cases (red line) generated by model for the years 2001–2011.

The results from model (2) indicated that maximum temperature, minimum temperature, rainfall, and adjusted relative humidity could possibly explain about 54% of the disease incidence; whereas, together with the past reported cases, the model explained about 80% of the distribution of the disease incidence. Model (2) generated fitted cases at SRMSE of 0.26 and R^2^ of 0.87. Residual analyses showed a fit with the model. Residual plots displayed patterns that suggested near normal, random, and linear data. The PACF of residuals indicated no auto-correlation with all the spikes within the upper and lower bands of the confidence intervals. A time series plot of fitted cases showed a satisfactory match against reported cases; thus, suggesting that the model was sufficient for interpretation (Figure 4).

**Figure 5:**
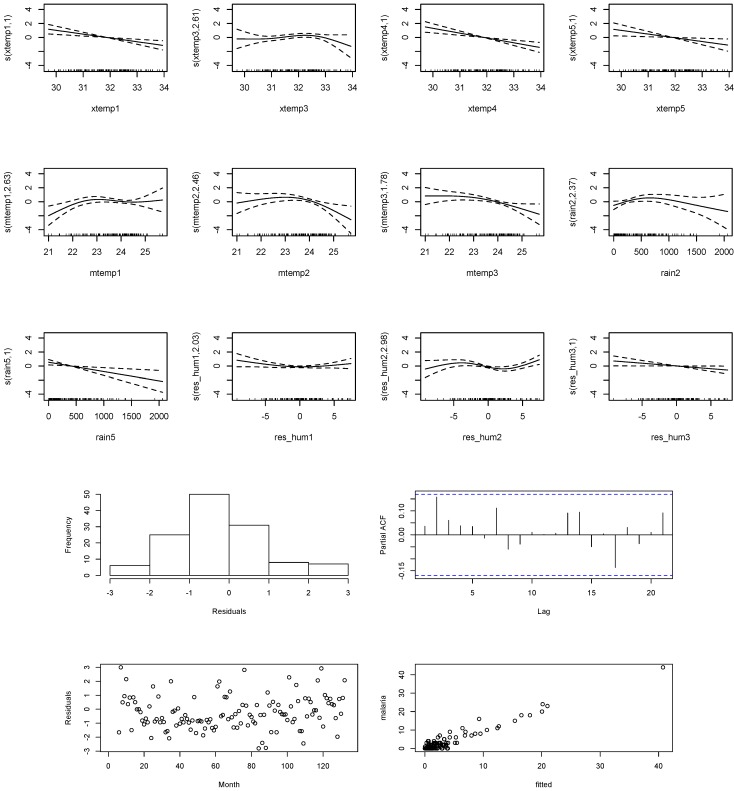
Relative risk of malaria cases as functions of respective climate variables at various lag times.

Figure 5 shows that maximum temperature potentially induced an increase in the relative risk of malaria at lag terms up to five months, though the risk level reduced with increasing temperature. Maximum temperature ranging from 29 °C to 32.5 °C corresponded to positive relative risks of malaria at lag of one, four, and five months. A maximum temperature exceeding 33 °C could possibly reverse the risk of malaria incidence in the study area. The relationship between the relative risk of malaria and minimum temperature at lag of 1–2 months could be presented as a bell shape. The risk increased as the minimum temperature rose from 22 °C, peaking at around 23.5 °C, and decreased thereafter. A unit decreased in minimum temperature from 25 °C to 21 °C corresponded to a rise in the relative risk of malaria at lag of three months. Rainfall preceded malaria by 2 and 5 months. Relative risk of malaria increased linearly at a lag of two months, given a unit rise of rainfall from 0 mm, with a peak at 500 mm, before declining with increasing rainfall. Rainfall from 0 mm to 500 mm posed a risk of malaria at a lag of five months, with diminishing risk levels as the amount of rainfall increased. Adjusted relative humidity exerted positive influence on malaria at a lag of one and two months, with an inverse relationship at a lag of three months.

## 4. Discussion

Over a period of eleven years (2001–2011), dengue showed the highest peak during the rainy season, especially in July. But malaria was mostly reported during the summer, with the highest peak occurring in March. Since both diseases varied seasonally, it can be concluded that variations in local climate could change and modify transmission patterns. Some changes could be beneficial for dengue transmission, whereas the same changes could have a negative impact on malaria transmission. Effective implementation of prevention and control measures adapted to deal with specific diseases, either dengue or malaria, in this tourist setting needs to be investigated.

Our study demonstrates positive associations between meteorological factors and reported incidences of vector-borne diseases such as malaria and dengue. It also identifies a potential predictive ability for these vector-borne diseases, which may help developing timely preparedness and intervention measures. The strengths of association between meteorological data and the reported cases of each disease varied according to the combined effects of climatic variables at various time windows. The impact of climatic factors on dengue or malaria could be compounded by numerous complex factors, ranging from the life cycles and characteristics of the vectors responsible for transmitting respective diseases to the evolution of the dengue virus or malaria parasites, to host immunity levels, to local vector control policies, healthcare structures, inter-sectorial collaborations, community commitment on the elimination of larvae, population lifestyles and water storage habits, building structures and maintenance, presence of water catchments, parks in the study area, and more.

Our findings showed that mean temperature, adjusted relative humidity, and rainfall preceded an increasing risk of malaria by up to 6–7 months. Generally, the highest relative risk of dengue corresponded to each unit increase in mean temperature, rainfall, and adjusted relative humidity at a lag of two, one and six months, respectively. Higher temperature, rainfall, and humidity tended to influence dengue incidence at longer lag periods. Relative risk of malaria increased up to five months subsequent to favorable climate conditions. Increase in the relative risk of malaria could be induced by a maximum temperature below 32 °C, minimum temperature below 24 °C, rainfall below 500 mm, and adjusted relative humidity with reduced effects from temperature and rainfall. Overall, low to moderate rainfall coupled with lower maximum temperature posed a threat for malaria at a lag period up to five months; while minimum temperature and adjusted relative humidity influenced malaria at a shorter lag period of three months.

In comparison, meteorological factors tended to influence dengue incidence on Koh Chang at a longer lag period when compared to malaria incidence. The effects of rainfall on dengue differed from malaria. Rainfall below 500 mm induced a higher risk to malaria after one month, whereas rainfall above 500 mm increased the risk of dengue after six months. At the same time, adjusted relative humidity affected malaria at lag terms up to three months, while it influenced dengue up to seven months. Different life cycles, characteristics, breeding habits, and ability to adapt to harsh environments could be speculated as partial reasons for these phenomena. However, further study is needed in order to shed light on these issues.

Our findings are consistent with other studies that have reported high dengue transmission during rainy seasons, a period with high rainfall and humidity that provides more breeding sites for mosquitoes, therefore increased transmission [14,15]. Our results were also in agreement with other studies that have documented high malaria transmission during or immediately after the rainy season [16,17] and during the dry season at a lag of five months [18]. The higher number of malaria cases occurring during the summer on Koh Chang might be explained by the practice of staying (or sleeping) outdoors during the summer or dry season without enough protective clothing, which enhances the risk of malaria occurrence [19]. As seasonal variations resulted from natural phenomenon or human activities, it might alter both dengue and malaria transmission in the future.

For dengue, the minimum and mean temperatures were positively correlated with dengue incidence. However, while maximum temperature, rainfall, and relative humidity were usually positively correlated with dengue incidence, in some periods, they appeared to show a negative correlation. For malaria, maximum temperature and mean temperature seem to positively correlate with malaria incidence, but in some periods, they appeared to show a negative correlation. Minimum temperature, rainfall, and relative humidity were mostly negatively correlated with malaria incidence. Positive correlations between dengue incidence and meteorological factors (minimum temperature, average temperature, and relative humidity) have been found on a monthly basis [20,21,22,23].

While relative humidity resulting from heavy rainfall might wash away mosquito larvae [24], some believe that mosquito larvae are affected only temporarily by heavy rainfall and that disease occurrence or transmission of malaria is still possible [25].

Meteorological factors (temperature, precipitation, humidity, and wind speed) as well as public health services affect the spread of the diseases [4]. They affect directly or indirectly humans and non-humans through altering their environments, which in turn influence social, economic and health conditions. The impacts are felt at local or national levels first, but could expand internationally. All sectors and responsible authorities should take this issue as a potential concern, and measures and actions at all levels should be promptly applied. However, other factors (*i.e.*, human factors: behavior, immunity, socioeconomic influences) might also contribute to the complexity of climatic and disease ecology and incidence. As the relationship between meteorological variables and factors that influence dengue transmission are complex, a true understanding of the whole system (ecological, biological and sociological aspects) should be applied and studied in detail in collaboration with all involved organizations from the bottom to top of the system.

## 5. Conclusions

We have, in this study, shown a relationship between meteorological and disease surveillance data (dengue and malaria) in the study island of Koh Chang. Disease transmission seasonally differed between dengue and malaria. Dengue occurred mostly in the rainy season, whereas malaria was predominantly present in the summer. These findings may be useful for assisting the implementation of effective prevention and control programs to prevent or decrease disease transmission.

Scientific evidence of the effects of climate on dengue and malaria could enhance mitigation efforts and reduce the public health burden. We encourage similar studies using quality data and higher time resolution to further analyze the effects of climate on malaria and dengue and to explore the possibility of forecasting dengue and malaria based on optimal lead times. Such information could empower policy makers to formulate strategic preventive measures and to make informed decisions on reducing the threat of widespread epidemics; especially when an epidemic on Koh Chang, being a global outreach tourism hotspot, could generate ripple effects at national and international levels.
